# Terminal Ileitis on Imaging Is Rarely Crohn's Disease: A 10‐Year Retrospective Cohort Study

**DOI:** 10.1002/jgh3.70459

**Published:** 2026-08-10

**Authors:** Irene Lu, Carl Cosgrave, Phongsakone Inthavong, Phillip Te, Zacchary Tamsett, Jacoba van Wees, Neel Heerasing

**Affiliations:** ^1^ Department of Gastroenterology Barwon Health Geelong Victoria Australia; ^2^ Department of Gastroenterology Northern Health Melbourne Victoria Australia; ^3^ Department of Infectious Disease Barwon Health Geelong Victoria Australia; ^4^ School of Medicine, Deakin University Geelong Victoria Australia

**Keywords:** biomarkers, Crohn's disease, hypoalbuminemia, inflammatory bowel disease, terminal ileitis

## Abstract

**Background:**

Terminal ileitis is a common radiological finding in patients with abdominal pain and altered bowel habits. Although often associated with Crohn's disease (CD), it has a broad differential diagnosis including infectious, surgical, and malignant conditions. Prior studies suggest 20%–45% of terminal ileitis accounts for CD. This study aimed to evaluate the proportion of patients with computed tomography‐detected (CT) terminal ileitis diagnosed with CD and to assess clinical and biochemical predictors.

**Methods:**

A retrospective cohort study was conducted at a single tertiary hospital between 2014 and 2024. Adult patients with CT findings of terminal ileitis were included. Data were collected on demographics, laboratory values, stool testing, and endoscopic evaluation. Multivariable logistic regression was used to identify predictors of CD.

**Results:**

Of 128 patients, 16 (12.5%) were diagnosed with CD. Infectious (27.7%) and surgical (17.9%) etiologies were the most common causes of non‐CD terminal ileitis. Stool testing within 72 h was performed for 48.4% of patients, with 35.5% positive for pathogens. Endoscopic evaluation was performed in 59 patients (46%), and histopathological inflammation identified in 43.5% of biopsied cases. Lower albumin levels trended toward association with CD and were independently associated with CD on multivariable analysis (OR 0.82, 95% CI 0.72–0.94, *p* = 0.004).

**Conclusions:**

After evaluation, a small minority (12.5%) of patients with CT‐detected terminal ileitis were diagnosed with CD. Hypoalbuminemia was a significant predictor of CD, whereas routine inflammatory markers had limited diagnostic value. Larger prospective studies are needed to further refine the diagnostic algorithms for detection of CD in patients with terminal ileitis.

## 1 Introduction

Terminal ileitis refers to inflammation of the distal ileum with radiological findings, endoscopic manifestations, and histological change [1]. Although classically reported as a hallmark of Crohn's disease (CD), terminal ileitis encompasses a wide range of differential diagnoses, including gastrointestinal infections, *Mycobacterium tuberculosis* (TB), Behcet's disease, medication‐related enteropathy such as nonsteroidal anti‐inflammatories (NSAIDs), acute appendicitis, and less commonly, small bowel lymphoma, neutropenic or radiation injury, and infiltrative conditions including sarcoidosis/amyloidosis [2, 3]. Prior studies suggest approximately 20%–45% of terminal ileitis accounts for CD, although this significantly varies by population characteristics and is higher in chronic symptomatic cases [1, 4]. Mild, asymptomatic ileitis is furthermore increasingly recognized—both on imaging modalities and routine endoscopy [5]. The implications of this remain debated, balancing the proportion of patients at risk of developing CD with more recent data suggesting better outcomes with early intervention, versus mislabelling benign, nonspecific ileitis [5, 6, 7].

CD is a chronic condition with rising world‐wide incidence, characterized by transmural, discontinuous inflammation that may affect any part of the gastrointestinal tract [8]. CD is a clinical diagnosis, supported by characteristic endoscopic, histological, and radiological features; sometimes requiring longitudinal follow up to confirm diagnosis and distinguish from infection or other etiology. Ileal involvement is a common finding, with the ileum affected in at least 70% of all CD [9]. Characteristic endoscopic features include focal ulcerations and segmental involvement, and histology may show chronic inflammation, with or without granulomas [10]. Occasionally, CD is diagnosed on surgical resection specimen, without endoscopy, when patients present with small bowel perforation or abscess.

Overall, terminal ileitis is a frequent radiological finding on computer tomography (CT), particularly in patients presenting with abdominal pain and altered bowel habits. Clinical guidelines recommend that in the absence of an alternative diagnosis (e.g., positive infective work up), terminal ileitis should be further evaluated with timely endoscopy, including terminal ileal (TI) intubation and biopsy [11]. Preliminary studies suggest a strong need to consider alternative diagnoses to CD when terminal ileitis is identified on imaging [12]. Stool testing is essential for excluding infectious etiologies; however, results often take several days to return due to laboratory processing times and batching protocols.

C‐Reactive Protein (CRP) is a well‐established objective biomarker of inflammation and correlates with disease activity in CD once the diagnosis has been established [12]. However, it is nonspecific and may also be markedly elevated in infectious processes and malignancies [13]. Fecal calprotectin (FCP) is a sensitive, noninvasive biomarker of intestinal inflammation and is widely used to monitor and evaluate inflammatory bowel disease (IBD); although similarly, is not disease specific [14]. Conversely, albumin is a negative acute‐phase reactant, with reduced levels often observed in inflammatory states, particularly in chronic conditions [15, 16]. Therefore, hypoalbuminaemia may help differentiate from more acute pathologies, such as infection or appendicitis. To date, few studies have specifically evaluated the predictive value of CRP and albumin for diagnosing CD in patients with CT‐detected terminal ileitis.

The aim of this study was to evaluate the proportion of patients with CT‐detected terminal ileitis who were subsequently diagnosed with CD. Secondary outcomes included characterizing alternative diagnoses and evaluating additional clinical and biochemical parameters associated with a subsequent diagnosis of CD from CT‐detected terminal ileitis. Specifically, serum albumin, CRP, FCP, the Charlson Comorbidity Index (CCI), smoking status, NSAID usage, nationality, and epidemiological risk for TB were evaluated as potential predictors of diagnostic outcomes.

## 2 Methods

We performed a retrospective cohort study of people who underwent CT abdomen with radiological findings of terminal ileitis at a single tertiary hospital during a 10‐year period between January 2014 and January 2024. The hospital is the largest regional hospital in Victoria, Australia, servicing a population of approximately 350 000 with all cases identified through its in‐house radiology service. This was done by searching all abdominal CT reports in the time period for the phrase “terminal ileitis,” after which each report was reviewed to confirm radiological features. Patients were excluded if they were younger than 18 years, had a known diagnosis of CD prior to the study period, or had insufficient medical documentation.

Data was systematically collected from patients records regarding age, sex, albumin, CRP, CCI, smoking status, NSAID usage, epidemiological risk for TB, and clinical indication for CT. Patients were followed longitudinally to document stool specimen testing, endoscopic evaluation including ileal intubation and subsequent final diagnosis, that is, CD or an alternative etiology (surgical, infectious, neutropenic, malignant, or other causes). For analysis, patients were grouped according to whether terminal ileitis was ultimately attributed to CD or a non‐CD cause.

Statistical analyses were performed using Stata version 18.0 (Basic Edition). Categorical variables were compared using the *χ* ^2^ test or Fisher's exact test as appropriate. Continuous variables were summarized using medians and compared using Mann–Whitney U test as they were evaluated as not normally distributed. Multivariable logistic regression was performed to examine the association between CRP, albumin, age, and CCI and the presence of CD. Model performance was assessed using the area under the receiver operating characteristic curve (AUC) and the discrimination slope. All tests were two‐sided, and a *p* < 0.05 was considered statistically significant.

## 3 Results

**FIGURE 1:**
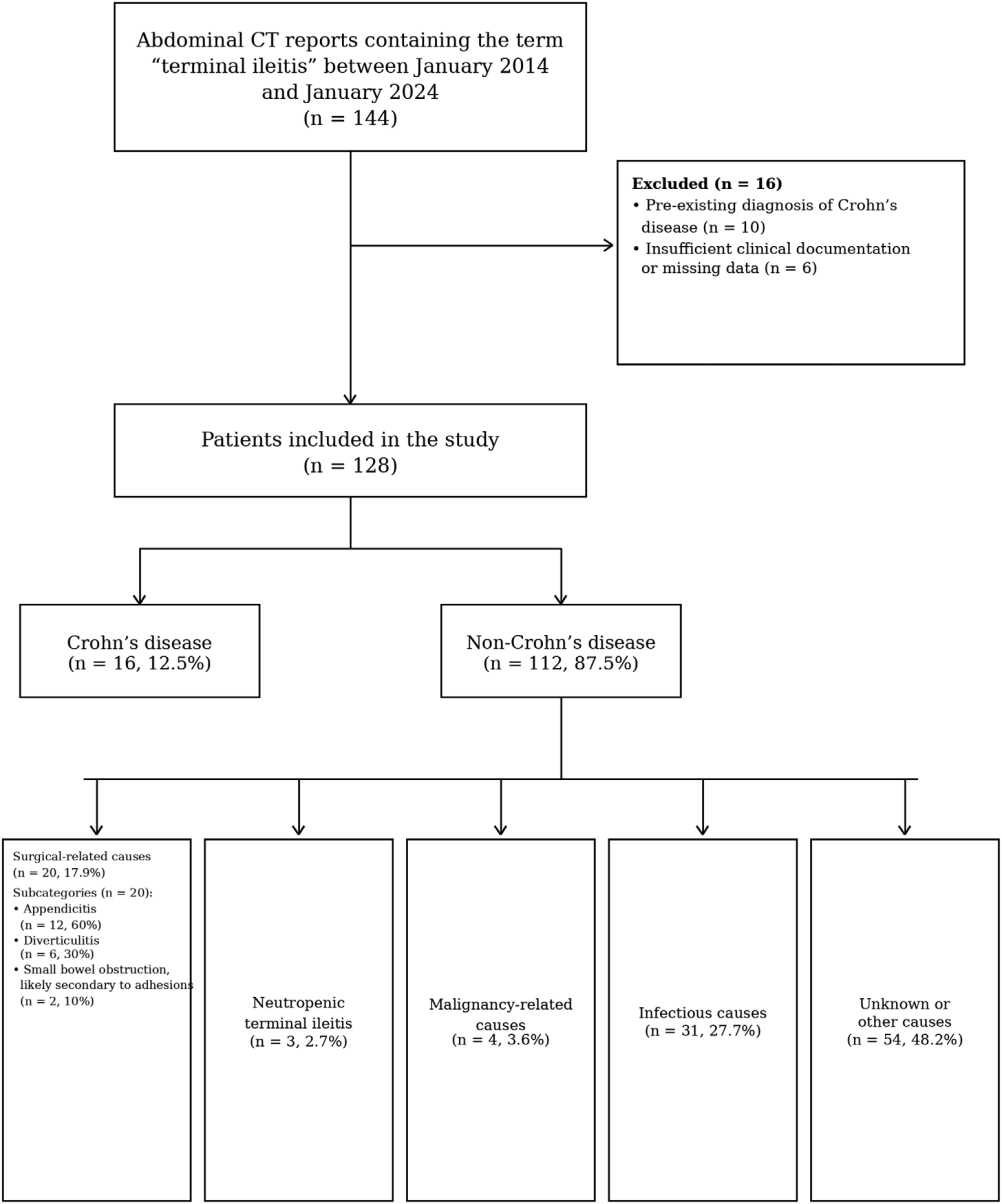
Flow diagram of patient selection and final diagnostic categories.

We initially identified 144 cases using the screening method but excluded 16 cases due to subsequent detection of previous CD diagnosis or missing data. The total number included in this study was 128. Of these, 112 (87.5%) were in the non‐CD group and 16 were in the CD group. The cases in the non‐CD group were 17.9% (20) surgical‐related causes, 2.7% (3) neutropenic‐terminal ileitis, 3.6% (4) malignancy‐related, 27.7% (31) infections, and 48.2% (54) unknown or other cause. Among the surgical cases, the majority were appendiceal or diverticular disease and associated perforation: 60% (12) involved appendicitis, 30% (6) were diverticulitis, and 10% (2) involved small bowel obstruction, likely secondary to adhesions, although further characterization was limited. Figure 1 demonstrates patient selection and final diagnostic category.

### 3.1 Baseline Characteristics

Baseline characteristics are summarized in Table 1. The median age of participants was 39.5 years (26.5–60). The cohort was evenly distributed by sex, with 65 females (50.8%) and 63 males (49.2%). The majority of patients were of Australian nationality (85.9%), while 18 (14.1%) patients had non‐Australian nationality. Of the 128 cases, five patients were classified as having a high epidemiological risk and one as having a moderate epidemiological risk for TB based on nationality, according to World Health Organization (WHO) data. No cases of TB‐related terminal ileitis were identified.

**TABLE 1:** Baseline demographics of the study cohort.

Demographics	Crohn's disease (*n* = 16)		Non‐Crohn's disease (*n* = 112)		All (*n* = 128)	
		IQR or %		IQR or %		IQR or %
Age, years (median)	40	(26.5–57)	40	(27–61)	39.5 years	26.5–60
Sex						
Female (*n*)	9	56.3	56	50.5	65	50.8
Male (*n*)	7	43.8	55	49.6	63	49.2
Smoking^a^						
Nonsmoker (*n*)	8	50	69	62.7	78	61.4
Current smoker (*n*)	6	37.5	15	13.6	32	25.2
Ex‐smoker (*n*)	2	12.5	15	13.6	17	13.4
NSAID use (*n*)	1	6.3	15	93.8	11	8.7
Nationality—Australian (*n*)	11	68.8	98	88.3	110	85.9
Serum ablbumin (median)^b^	30.5 g/L	30–35	36	32–39	35 g/L	31–39
CRP (median)^c^	52 mg/L	20.1–136	63.9 mg/L	17.1–140	56 mg/L	18.3–137

^a^ Smoking status was missing for one patient and excluded from percentage calculations.

^b^ Serum albumin values were available for 120 of 128 patients.

^c^ CRP values were available for 117 of 128 patients.

The indication for performing CT was most often due to gastrointestinal symptoms, including abdominal pain (104/128, 81.3%) and altered bowel habit and/or rectal bleeding (38/128, 29.7%). Other indications were present in 15 patients (11.7%), and presenting indications were not mutually exclusive. Presenting indications for CT are summarized in Table 2.

**TABLE 2:** Clinical indication for initial CT detecting terminal ileitis.

Presenting indication for CT^a^	*n* (%)
Abdominal pain	104 (81.3)
Altered bowel habit (including rectal bleeding)	38 (29.7)
Other clinical indications^b^	15 (11.7)

^a^ Patients could have more than one indication for CT; therefore, percentages exceed 100%.

^b^ Other indications included cancer staging, weight loss, fever, obstructive symptoms, follow‐up imaging, postoperative pain, and other miscellaneous clinical presentations.

### 3.2 Endoscopy

Of all patients, 59 (46%) proceeded to endoscopy, with associated histopathological findings summarized in Table 3. A total of 51 (87.9%) had TI intubation and 46 of these had biopsies taken of the TI. In 20 cases (43.5%) histopathology demonstrated inflammatory changes (IBD‐specific or nonspecific), while in another 24 (52.2%) cases, no inflammation was seen and two were inconclusive due to difficult histological read and diathermy artifact. Among the patients who underwent endoscopic evaluation, 11 (18.6%) were subsequently diagnosed with CD while 48 (81.4%) had non‐CD causes of terminal ileitis. An additional five patients were diagnosed with CD despite not having endoscopy recorded. Of these, three3 were diagnosed based on histopathological examination of surgical resection specimens, and two underwent diagnostic endoscopy at external institutions with their CD diagnosis confirmed through review of gastroenterology follow‐up documentation. The median time from CT to endoscopy was 7 weeks (IQR 4–16).

**TABLE 3:** Endoscopic evaluation and histopathological findings.

Diagnosis pathway	*n* (%)
Total cohort	128 (100)
Endoscopy	
Patients undergoing endoscopy	59 (46.1)
Terminal ileum intubated	51 (87.9)
Terminal ileal biopsies obtained	46 (90.2)
Terminal ileal histopathology (*n* = 46)	
Any inflammation (IBD or nonspecific)	20 (43.5)
IBD‐specific inflammation	5 (10.9)
Nonspecific inflammation	15 (32.6)
No inflammation	24 (52.2)
Inconclusive	2 (4.3)
Final diagnosis among patients who underwent endoscopy (*n* = 59)	
Crohn's disease	11 (18.6)
Non‐Crohn's disease	48 (81.4)

### 3.3 Stool Testing and FCP

Stool testing using microscopy, culture, and sensitivity (MCS) and/or viral polymerase chain reaction (PCR) was performed within 72 h for 62 of the 128 cases (48.4%). Of these, 22 (35.5%) returned a positive result, with 19 (30.6%) detected a pathogen. There were 16 cases of *campylobacter*, two cases of *salmonella spp*, one case of *dientamoeba fragilis*, one case of *norovirus*. *Blastocystis hominis* was detected in four cases and was not considered to be pathogenic.

FCP was measured in 21 cases (16.4%) and the median result was 154 μg/g (IQR 62–412), with mean of 1028 μg/g. The median FCP was 152.5 μg/g (IQR 62–315) for cases without CD (*n* = 18), and 800 μg/g (7–10 500) for cases with CD (*n* = 3); the difference was not statistically significant (*p* = 0.44).

### 3.4 Univariate Analysis

#### 3.4.1 Age and Sex

There was no difference in age for patients diagnosed with CD or without CD. However, younger age was associated with a higher likelihood of TI intubation (*p* = 0.017); median age was 37 years (IQR 28–50) in the group with TI intubation and 61 years (IQR 33–68) without TI intubation. Sex was not associated with CD diagnosis (*p* = 0.66) and did not influence progression to endoscopy (*p* = 0.73) or histopathological findings.

#### 3.4.2 Albumin

Albumin tended to be lower in patients diagnosed with CD (30.5 g/L, IQR 30–35) compared to the non‐CD group (36 g/L, IQR 32–39); however, this difference was not statistically significant on univariate analysis (*p* = 0.063). Meanwhile, albumin levels appeared higher in infectious ileitis (34 g/L IQR 32–39) and in surgical causes for ileitis (34 g/L IQR 30.5–37). Using the local laboratory reference cut off value of 35 g/L, hypoalbuminaemia was present in 43% of patients. Categorization of albumin into normal range **≥** 35 g/L versus low range albumin < 35 g/L did not significantly alter the findings. Median serum albumin across patient subgroups is summarized in Table 4.

**TABLE 4:** Serum albumin by diagnostic subgroup.

Subgroup (*n*)	Median albumin, g/L (IQR)	Subgroup (*n*)	Median albumin, g/L (IQR)	*p*
Crohn's disease (16)	30.5 (30–35)	Non‐Crohn's Disease (104)	36 (32–39)	0.063
Infectious etiology (29)	34 (32–39)	Noninfectious etiology (91)	35 (30–39)	0.75
Surgical etiology (20)	34 (30.5–37)	Nonsurgical etiology (100)	35.5 (31–39)	0.16
Endoscopy performed (57)	37 (32–29)	Endoscopy not performed (62)	33 (30–38)	0.22

*Note:* Albumin and CRP were measured within 72 h of CT. Continuous variables were analyzed using Mann–Whitney U tests and univariable logistic regression. Categorized analyses used *χ* ^2^ testing. Values are reported as median.

#### 3.4.3 CRP

CRP was not associated with a diagnosis of CD in patients with CT‐detected terminal ileitis. However, the CRP trended higher in patients with histopathological evidence of IBD (*p* = 0.06). Higher CRP levels were significantly associated with infectious (*p* = 0.003) and surgical etiologies (*p* = 0.01) and also determined whether there was progression to endoscopic evaluation (*p* < 0.001), as shown in Table 5. Categorizing CRP into low (< 10 mg/L) and high (≥ 10 mg/L) values did not substantially alter these associations (infection vs. noninfection, *χ* ^2^ test, *p* = 0.012).

**TABLE 5:** Comparison of median CRP across patient subgroups.

Subgroup (*n*)	Median CRP, mg/L (IQR)	Subgroup (*n*)	Median CRP, mg/L (IQR)	*p*
CD (14)	52 (20.1–136)	Non‐CD (103)	63.9 (17.1–140)	0.79
IBD (4)	109 (50.5–201.5)	Not IBD (36)	28.7 (14.3–74.15)	0.06
Infectious etiology (30)	108.5 (50.7–170)	Noninfectious etiology (87)	48 (13.5–120)	0.003
Surgical etiology (20)	130.5 (45.3–181)	Nonsurgical etiology (97)	50.7 (16–116)	0.01
Endoscopy performed (53)	47 (13.8–76.8)	Endoscopy not performed (63)	113 (21.4–170)	< 0.001

#### 3.4.4 Charlson‐Comorbidity Index (CCI)

Overall CCI scores were low with 56% of cases having a CCI of 0. CCI was not associated with a diagnosis of CD (*p* = 0.75). Higher CCI was associated with a reduced likelihood of TI intubation (*p* = 0.02). The distribution of CCI by etiology is summarized in Table 6.

**TABLE 6:** Charlson Comorbidity Index (CCI) distribution by etiology.

CCI score	Etiology		Total (*n*)
	Non‐CD (*n*)	CD (*n*)	
0	62	8	70
1	11	3	14
2	8	2	10
3	9	0	9
4	5	0	5
5	4	0	4
6	5	1	6
7	1	0	1
8	1	1	2
9	2	0	2
10	2	0	2

#### 3.4.5 Smoking

Smoking status was not associated with a diagnosis of CD, histological inflammation, or endoscopic outcomes, including progression to endoscopy and TI intubation. In contrast, infectious terminal ileitis was significantly more common among nonsmokers compared with current or ex‐smokers (*p* = 0.010). This association remained significant when smoking was analyzed as ever‐smoker (ex‐smokers and current) versus life‐long nonsmokers. (*p* = 0.003).

#### 3.4.6 Nonsteroid Anti‐Inflammatory Drugs (NSAIDs)

NSAID use was uncommon in the cohort and was not associated with CD, histological inflammation, infectious or surgical etiology, or endoscopic outcomes, including progression to endoscopy, TI intubation, or biopsy performance (all *p* > 0.05).

### 3.5 Multivariate Analysis

Multivariable logistic regression analysis was performed using parameters of CRP, albumin, age, sex and CCI which was selected on the basis of clinical relevance. While albumin levels trended lower in CD than non‐CD (median 30.5 vs. 36 g/L) on univariate testing, multivariate analysis demonstrated that lower albumin level was independently associated with CD with statistical significance (OR 0.82, 95% CI 0.72–0.94, *p* = 0.004). Other variables were not significant predictors. The exploratory multivariable model demonstrated an apparent AUC of 0.80.

## 4 Discussion

In this study, we demonstrate that the likelihood of CD among patients with CT‐detected terminal ileitis is low, with only 12.5% subsequently receiving a diagnosis of CD. Infectious (27.7%) and surgical‐related (17.9%) causes were the most common identified etiologies, although nearly half of the cases (48.2%) remained of unknown origin. A substantial proportion of these are likely attributable to undetected infection, given that more than 50% of patients did not complete stool sampling within 72 h, combined with the known limited sensitivity of fecal MCS, as well as the lack of bacterial PCR based testing at this study site [17]. These findings are consistent with prior observational data, including a study by Pais et al., in which only 32% of patients with radiological terminal ileitis were diagnosed with CD, while 62% had an infectious cause [12].

The relatively high proportion of surgical‐related pathology underscores the importance of considering acute surgical conditions, particularly appendicitis, where delayed recognition may result in perforation or significant morbidity [18]. Other etiologies, including neutropenic ileitis and malignancy, were uncommon, and no cases of TB were identified. This likely reflects the non‐TB‐endemic setting of our cohort and would differ in regions with higher TB prevalence or greater migrant populations, given TB can closely mimic CD both clinically and radiologically [19].

Demographic analysis did not identify younger age as a predictor of CD, in contrast to previous studies [20]. This may reflect reduced discriminatory power, as our cohort was relatively young overall (median age 39.5 years). No sex‐based differences were observed, consistent with existing literature, although prior studies such as Bermont et al. have reported male predominance, likely representing cohort‐specific variation [16, 20].

Given the diagnostic challenges associated with CD, several studies have sought to identify clinical, biochemical and radiological predictors to facilitate earlier diagnosis [4, 20, 21]. Shen et al. developed a clinical prediction model for patients presenting with abdominal symptoms in primary care, demonstrating that combinations of demographic characteristics, symptom profile and routine laboratory parameters improved diagnostic discrimination compared with individual variables alone [4]. In TB‐endemic settings, Sachdeva et al. proposed an algorithm integrating clinical presentation, inflammatory markers, cross‐sectional imaging and endoscopic findings to distinguish CD from other causes of terminal ileitis, particularly intestinal TB [20]. More recently, Bermont et al. evaluated patients with CT‐detected terminal ileitis and identified male sex, abdominal pain and lower mean corpuscular volume (MCV) as independent predictors of CD, although the authors acknowledged that the predominance of male patients in their cohort may have influenced these findings [21].

Our study builds on this literature by examining an unselected, real‐world cohort of patients with CT‐detected terminal ileitis in non‐TB‐endemic setting. We evaluated a comprehensive range of demographic (age, sex, nationality), clinical (CCI, smoking status, NSAID use), and biochemical (albumin, CRP, FCP) variables.

In contrast to previous studies, neither younger age nor male sex independently predicted CD in our cohort. Instead, hypoalbuminemia emerged as the only independent predictor of subsequent CD diagnosis. This association is biologically plausible, reflecting the chronic inflammatory state, intestinal protein loss and nutritional impairment found in CD. This contrasts infective and reactive causes of terminal ileitis, which is typically acute and less likely to result in sustained reductions in serum albumin. As a readily available and inexpensive biomarker, serum albumin therefore provides incremental value when combined with other clinical, laboratory and radiological variables during the initial assessment of patients with CT‐detected terminal ileitis.

Taken together, the available evidence suggests that no single predictor is sufficiently accurate to diagnose CD in isolation. Rather, future prospective multicentre studies should evaluate combinations of predictors identified across existing studies. Such models may facilitate earlier gastroenterology referral, prioritization for ileocolonoscopy and more efficient allocation of diagnostic resources.

Although CRP was not independently associated with a diagnosis of CD, levels trended higher in patients with histopathological features consistent with IBD (*p* = 0.06). Elevated CRP may reflect overall inflammatory burden rather than disease etiology, given its lack of specificity and elevation in infectious and acute surgical conditions. As such, CRP may be more useful as a longitudinal marker of disease activity than as a discriminator of CD.

Several limitations should be acknowledged; the retrospective, single‐center design introduces the potential for selection bias and incomplete data capture. In particular, incomplete stool testing may have led to underestimation of infectious causes. Additionally, the relatively young age distribution of the cohort may have limited the ability to detect age‐related associations. Possible verification bias occurred as not all patients underwent endoscopic evaluation. Although follow‐up clinical records were reviewed to ascertain subsequent diagnoses, it remains possible that some cases of CD were not identified. The multivariable model has low events‐per‐variable ratio due to smaller numbers of CD events and inclusion of five covariates (age, sex, CRP, albumin and CCI). These variables were selected on clinical relevance to account for clinically important confounders, rather than statistical significance. Consequently, the model may be susceptible to overfitting and unstable coefficient estimates. Given the limited number of CD events, no formal internal validation was performed, and the apparent AUC may overestimate the model's performance. Therefore, these findings should be interpreted as exploratory and require validation in larger cohorts.

Future prospective, multicenter studies—including cohorts from TB‐endemic regions—are required to validate these findings. Building on predictors identified across existing studies, future models should evaluate combinations of demographic (age and sex), clinical (symptom duration and abdominal pain), biochemical (serum albumin, CRP, FCP, and hematological indices including MCV and platelet count) and CT imaging variables. External validation of such models will be essential before they can be incorporated into routine clinical practice.

This study demonstrates that CD accounts for a minority of cases of CT‐detected terminal ileitis (12.5%), with infectious and surgical etiologies being more common. Hypoalbuminemia was independently associated with CD and may aid in risk stratification. Prospective, multicenter studies are warranted to validate these findings and to develop predictive models to facilitate earlier diagnosis.

## Funding

The authors have nothing to report.

## Ethics Statement

This study was approved to commence by the Barwon Health Human Research Ethics Committee. The study met requirements under the National Statement on Ethical Conduct in Human Research (NHMRC, 2023) and the Australian Code for the Responsible Conduct of Research (NHMRC, 2018). It is consistent with Ethical Considerations in Quality Assurance and Evaluation Activities (NHMRC, 2014).

## Conflicts of Interest

The authors declare no conflicts of interest.

## Data Availability

The data that support the findings of this study are available from the corresponding author upon reasonable request.
